# Advancing diagnostics for Chagas disease: key product characteristics and harmonized evaluation strategies - an expert meeting report

**DOI:** 10.1186/s12879-026-13565-3

**Published:** 2026-06-17

**Authors:** Laura C. Bohorquez, Alejandro G. Schijman, Freddy Perez, Hector Coto, Andrea Marchiol, Maria-Jesus Pinazo

**Affiliations:** 1 FIND, Chemin du Pommier 40, Le Grand Saconnex, Geneva, 1218 Switzerland; 2 INGEBI-CONICET, Laboratorio de Biología Molecular de la Enfermedad de Chagas, Instituto de Investigaciones en Ingenieria Genetica y Biologia Molecular, Dr Hector Torres, Vuelta de Obligado 2490, Buenos Aires, 1428ADN Argentina; 3 Communicable Diseases Prevention, Control, and Elimination Department, Pan American Health Organization, Washington, DC USA; 4 Federal University of Health Sciences of Porto Alegre (UFCSPA), Porto Alegre, RS Brazil; 5 DNDi Drugs for Neglected Diseases initiative-Latin America, Rua São José 70, sala 601, Rio de Janeiro, Brazil; 6 CIBERINFEC, ISCIII - CIBER de Enfermedades Infecciosas, Instituto de Salud Carlos III, Madrid, Spain

**Keywords:** Expert recommendations, Consensus meeting, *Trypanosoma cruzi*, Point-of-care tests, Cost-effectiveness

## Abstract

Chagas disease is a global public health issue, life-threatening parasitic disease, affecting mostly underserved communities in Latin America. Yet, 10% of infected individuals are diagnosed, and 1% receive etiological treatment. Priority actions for disease elimination by 2030 are streamlining diagnostic methods and evaluating and developing point-of-care diagnostics. We convened 44 researchers, test developers, and health authorities, to provide scientific advice and reach consensus on *(i)* a harmonized protocol for evaluation of rapid diagnostic tests; *(ii)* preferred molecular diagnostics characteristics (scope, performance, operational features, pricing, and evaluation standards); and *(iii)* economic impact evidence needed to have a transformational impact in primary healthcare. We considered global and national perspectives with discordant evidence from multiple disciplines. Our outcomes provide a reference on practical applications and recommendations to promote test development and evaluation. Finally, we debate about the appropriate approach moving forward to meet the diagnostic gap, and propose how investments could be stimulated.

**Supplementary Information:**

The online version contains supplementary material available at 10.1186/s12879-026-13565-3.

## Introduction

Approximately, seven million people, mostly in Latin America, are infected with *Trypanosoma cruzi*, the parasite responsible for Chagas disease (CD), and 70 million are at risk of infection globally. Every year, over 10,000 CD-related deaths are reported, and the disease burden exceeds USD 690 million in healthcare costs and USD 8 billion in annual economic losses [1]. CD is primarily a chronic, silent condition, with fewer than 10% of infected individuals diagnosed and 1% receiving etiological treatment [2, 3].

Barriers to healthcare access include a cumbersome and costly diagnosis process; the limited availability of diagnostics at primary health centers; and the lack of integration of diagnostic practices into maternal and child health policies. Importantly, infection is curable if treatment is initiated soon after infection. In chronically infected patients, antiparasitic treatment can prevent mother-to-child transmission and potentially mitigate disease progression [4, 5].

Despite the availability of new point-of-care (POC) diagnostics, their use remains limited. The Pan-American Health Organization (PAHO) and national guidelines in endemic countries currently recommend serological rapid diagnostic tests (RDTs) only for screening and research purposes. Meanwhile, molecular tests are part of the national strategy in some endemic countries (e.g. Argentina and Chile) [6, 7], and non-endemic high-income countries (such as Spain, Switzerland and the United Kingdom) [8–10].

As part of the transition from disease control to elimination, the World Health Organization (WHO) has outlined priority actions in its 2030 road map targets for Neglected Tropical Diseases (NTD) [11]. For CD, these recommendations emphasize streamlining diagnostic methods and advancing the evaluation and development of POC diagnostic tests. Independent evidence on the performance and economic impact of new diagnostics is being generated by various institutions.

While PAHO/WHO has outlined broad diagnostic goals, we convened a scientific meeting with 44 experts focused on actionable, technical consensus for RDT validation, molecular test standards, and cost-effectiveness frameworks, addressing gaps left by high-level diagnostic guidelines.

This report summarizes the meeting’s proceedings, consensus reached, and its broader implications.

## Methods

The meeting was held on May 6 and 7, 2024, in Buenos Aires, Argentina, convening 44 experts, and 17 observers, including global researchers, non-for-profit test developers, public health authorities, and PAHO representatives (Appendix 1 SI). Participants were invited in their independent capacity (not as organizational representatives) and selected based on their expertise on the three focus areas (RDT evaluation, molecular tests, cost-effectiveness), geographical diversity and gender balance. Prior to acceptance, experts reviewed the agenda, and the meeting objective of reaching consensus on three key subjects:

1. **Harmonized Protocol to Evaluate RDTs**: To ensure the implementation of high-quality, comparable studies and to generate conclusions with greater evidence-based weight.
2. **Key Product Characteristics and Quality Standards of Molecular Tests**: To assess and guide test development and use for early diagnosis of *T.cruzi* infection.
3. **Cost-Effectiveness and Economic Impact**: To define the evidence needed for the integration of new diagnostics into health systems in Latin America.

Two chairpersons per focus subject were appointed, and three rapporteurs documented discussions.

### Pre-meeting survey engagement

In April, 2024, experts received the harmonized research protocol for the evaluation of RDTs (developed by PAHO and INS Chile with input from PAHO-selected expert), and a pre-meeting survey with 45 statements/questions (12 on RDTs; 33 on molecular tests). Participants rated agreement (5-point Likert: *fully agree*,* mostly agree*, *neither agree or disagree*,* somewhat disagree*, or *disagree*) and provided comments when they disagreed.

### Day 1. Expert presentations and plenary discussions

Aggregated survey results were presented, prioritizing topics with neutral/disagreed responses for deeper discussion.

#### Harmonized protocol to evaluate RDTs

PAHO presented the findings of the scoping review conducted in April 2024, for RDTs for *T. cruzi* infection in humans (Spanish and English studies from 1990 to 2023, reporting sensitivity and specificity data. Out of 247 identified articles, 30 met the inclusion criteria by four experts. An overview of the 23-section protocol, and its development process was presented.

#### Key product characteristics and quality standards of molecular tests

Experts provided an overview of the state-of-the art of molecular diagnostic methods, LAMP, Real Time PCR (qPCR), and other POC molecular tests that could be potentially adapted for *T. cruzi* detection, as well as controls and standards necessary for test evaluation.

#### Cost-effectiveness and economic impact

Experts outlined the pathways of diagnosis and care and challenges associated with the current diagnostic methods. Additionally, examples of cost-effectiveness analyses, budget impact analysis (BIA) and social costs related to health interventions that have been conducted to inform policy makers were presented.

### Day 2. Consensus reached and conclusions

Three working groups, 12–18 experts each, convened to finalize consensus on each of the three relevant subjects, harmonized protocol for evaluation of RDTs (Group 1), key product characteristics and quality standards of molecular tests (Group 2), and cost-effectiveness and economic impact (Group 3). Conclusions were presented in plenary.

### Post-meeting outcomes

In June, 2024, Group 3 finalized the cost-effectiveness sub-study into the harmonized research protocol for the evaluation of RDTs, in collaboration with PAHO and FIND.

The harmonized RDT protocol was published by PAHO as the first standardized guideline for CD RDT evaluation [12].

## Results and discussion

### Pre-meeting survey engagement

A total of 57% of the experts (25 out of 44) submitted their feedback through the pre-meeting survey. Most experts agreed with all statements regarding both the evaluation of RDTs and key product characteristics of molecular tests, with over 50% indicating they “*fully agreed*” or “*mostly agreed*” with each proposed statement. This strong preliminary consensus helped focus the subsequent in-person discussions on areas needing clarification.

#### Consensus on the harmonized protocol to evaluate RDTs

The scoping review identified 41 RDTs, of which 25 were commercially available. Reported sensitivities ranged from 90.1% to 100% (mean 94%), while specificities ranged from 95.5% to 100% (mean 98.5%). The draft protocol had initially proposed target threshold of 92% sensitivity and 95% specificity.

However, experts presented data from four recent independent evaluations of up to 11 RDTs, conducted with autochthonous samples in Argentina (Se 92–100%; Sp 76–96%); Bolivia (Se 62–98%; Sp 78–100%); Brazil (Se 93–100%; Sp 78–92%); and Colombia (Se 75–99%; Sp 71–100%) [13–16].

To note, the results of the independent evaluations partially aligned with findings from the scoping review. Probably the majority of published studies, and included in the scoping review, have not been conducted using autochthonous populations (country-specific), as the origin of the samples (circulating Discrete Typing Units - DTUs) is usually not reported. Next, RDT performance was prioritized for deeper discussions and to reach consensus on expected values for the harmonized protocol.

The pre-meeting survey results, and consensus reached during the meeting, about the harmonized protocol to evaluate RDTs are shown in Table 1 and Appendix 2 SI.

**Table 1:** Main results related to the harmonized protocol to evaluate RDTs (see also Appendix 2 SI)

Pre-meeting Survey		During the meeting
Consensus (> 50% agree)	**Statement ** ***(Do you agree on including/changing in the protocol the following?*** **)**	**Consensus Reached**
And < 15% neutral or disagree	**Structure**	**Purpose.** Specify that the protocol focuses on performance evaluation of individual RDTs in the field compared to the reference method, not for analytical performance evaluation (remove “inclusion of samples with varying antibody levels”). Clearly separate two sections, one for laboratory performance evaluation, and another for field performance evaluation.
And < 15% neutral or disagree	**Expected performance.** Se/Sp and sample size	**Performance for Selection of Investigational Products (RDTs).** Recommend preselecting RDTs that reported previously a Se of 92% and Sp of 90% within a 5% full-width margin of error (95% confidence interval -CI +/- 2.5%). Prioritize primarily studies that evaluated RDTs with samples from autochthonous populations (country of interest). **Performance for Test Acceptance.** For given RDT(s), the expected lower bound is 92% for Se, and 95% for Sp; report the margin of error (95% CI). These parameters are used for sample size estimations. Prioritize conducting the study with autochthonous populations. **Include Sample Size.** To calculate the number of confirmed positive/negatives by the reference test method needed to estimate the expected Se/Sp (Appendix 3 SI). An online interactive tool is available <https://finddx.shinyapps.io/SampleSize/>
And 15–32% neutral or disagree	**Testing algorithms.** For hard-to-reach populations prioritize combining two RDTs with a tie-breaker	State that the purpose is to evaluate individual RDTs, particularly in the algorithm workflow section. However, consensus on performance for test acceptance can be used to evaluate diagnostic algorithms combining tests.
And 15–32% neutral or disagree	**Reference Test Method**	Clearly define it with a panel of reference validated tests with high Se/Sp values in the country. Recommend following the PAHO diagnostics guideline (2018) (agreement of at least two serological tests). This ensures comparability of a given RDT across the region.

#### Consensus on key product characteristics of molecular tests

The summary of expert presentations outlining the key requirements for new molecular diagnostics that can have a transformational impact, especially in primary healthcare facilities, and challenges for development and evaluation are summarized in Appendix 4 SI.

Main insights regarding key product characteristics, including the pre-meeting survey results, prioritized topics for plenary discussion, and to reach consensus during the meeting, for POC tests are displayed in Table 2, and for qPCR in Appendix 5 SI.

**Table 2:** Results about the key product characteristics of POC molecular tests

Pre-meeting Survey				During the meeting
Consensus (> 50% agree)	**Product Characteristic**	**Minimum Requirement**	**Ideal Requirement**	**Consensus Reached**
And < 15% neutral or disagree	Intended Use	Diagnosis for suspected acute infection (all transmission modes or infection reactivation)	Diagnosis for suspected acute infection (all transmission modes or infection reactivation); Asymptomatic, or symptomatic suspected chronic infection; Assessment of response to antiparasitic treatment	To recommend the inclusion of molecular methods in diagnostic algorithms, experts consider is necessary to generate more evidence to validate the available technologies, LAMP and qPCR, in the context of mother-to-child transmission, and on acute oral transmission outbreaks.
And < 15% neutral or disagree	Target Operator	Biochemist technician with basic training in molecular techniques	Not required	Unchanged
And < 15% neutral or disagree	Target Use Setting	Low basic lab / Second Level (DNA clean bench, heater device)	Primary care / Community testing	Unchanged
And < 15% neutral or disagree	Target Analyte	*T.cruzi* DNA	Multiplex (including the diagnosis of different pathogens)	Multiplex including internal amplification controls. Diagnosing in a multi-pathogen context would be ideal (perinatal HIV, syphilis and hepB), but it involves trade-offs with costs. Now, it is not a minimum requirement due to insufficient clinical-epidemiological evidence of co-morbidity with other diseases.
And < 15% neutral or disagree	Diagnostic Sensitivity	≥ 92% (point estimate with maximum +/- 95% CI) higher than microscopy and comparable to qPCR (S1 App 5)	≥ 95% (point estimate with maximum +/- 5% -95%CI) higher than microscopy and comparable to qPCR (S1 App 5)	Experts request PAHO/WHO to perform a systematic review of Se and Sp ranges based on current evidence considering regional epidemiological diversity.
And < 15% neutral or disagree	Reference Method	Microscopy / Standard algorithm for vertical infection		Use available field-validated molecular diagnostic kits as reference test method. Conduct validation studies in multicenter trials using autochthonous samples. Define the gold standard based on the clinical-epidemiological context: parasitological and serological methods for congenital cases, direct parasitological methods for other cases.
And < 15% neutral or disagree	Analytical Specificity	No cross-reactivity with other *Trypanosoma spp.*,* Leishmania spp*., or other pathogens		Unchanged
And < 15% neutral or disagree	Strain Specificity	Detects all DTUs		Unchanged
And < 15% neutral or disagree	Quantitation	No	Yes	Unchanged
And < 15% neutral or disagree	Training Needs	2–5 days training	Not required	Unchanged
And < 15% neutral or disagree	Specimen Type	< 500 µL anticoagulated whole blood (WB) / <125 µL in dried blood spots (DBS)	< 30 µL anticoagulated WB / A drop in DBS	Ideal samples are anticoagulated WB or DBS, compatible with DNA extraction kits. Use stabilizing solutions like Guanidine Hydrochloride 6 M, EDTA 0.2 M, pH 8.00 (GE) for transporting samples, following national guidelines. Validation studies tailored to specific clinical sample types and collecting/storage conditions (e.g. frozen EDTA-blood, plasma, buffy coat) are necessary. WHO/PAHO-driven validations should generate evidence on multiple sample collections, and DNA extraction procedures.
And < 15% neutral or disagree	Specimen Preparation	Rapid DNA extraction, a single replicate	Fully integrated, automated device	
And < 15% neutral or disagree	Processing Steps	No centrifugation, no pipetting needed during DNA extraction	Faster, near-instant results	Unchanged
And < 15% neutral or disagree	Need for operator to transfer a precise volume of sample	Yes	No	Unchanged
And < 15% neutral or disagree	Time sample collected to result	Five hours (40–50 min aplification)	< Two hours	Unchanged
And < 15% neutral or disagree	Power Requirements	Power 110/220 W	No electricity needed / Portable batteries / Solar energy	Unchanged
And < 15% neutral or disagree	Operating characteristics	Operating at up to 25 °C	Data analysis. Connectivity. Results capture and display. Operating at > 25 °C	Unchanged
And < 15% neutral or disagree	Internal Quality Control	Positive control, non-template control, and negative DNA extraction control included in kit	Quantitative positive control included in kit	Unchanged
And < 15% neutral or disagree	External Quality Control	Standard (third-party) reference panels	International certified harmonized (third-party) panels. And prospective field studies with blind samples	Experts request PAHO/WHO to promote the production of calibrators, international reference standards, and quantitative positive controls, developed regionally for different clinical-epidemiological contexts.
And 16–28% neutral or disagree	Diagnostic Specificity	Comparable to microscopy and qPCR		Consensus could not be reached on this regard. More evidence is needed from field studies to determine these values.
And 16–28% neutral or disagree	Analytical Sensitivity	One eq. parasite/ml in blood; 20 eq. par./ml in DBS	0.1–0.5 eq. par./ml	Use validated sequences for quantifying copy numbers in multicenter studies.
And 16–28% neutral or disagree	Specimen Capacity and Throughput	Up to 8 samples per run	> 8 samples per run	Unchanged
And 16–28% neutral or disagree	Instrumentation Integration	Simple reading device	Instrument-free (naked eye)	Unchanged
And 16–28% neutral or disagree	Reagent kit storage and stability	-20 °C, 6 months	About 25 °C, 18 months	Unchanged
And 16–28% neutral or disagree	Quality Assurance (QA)	Proficiency testing panels evaluated before starting implementation of a new assay, and every two years thereafter.	Proficiency testing panels evaluated every year.	Perform QA processes when instruments, reagent batches, or operators change. Follow strictly the instructions of manufacturers with operational controls included in the kits. Evaluate proficiency testing panels before implementing new assays one panel per year.
And 16–28% neutral or disagree	Test Price	Less than 10 USD	Less than 5 USD	Unchanged
And 16–28% neutral or disagree	Instrument Price	Less than 10,000 USD	Less than 1,500 USD	Unchanged

#### Conclusions on cost-effectiveness and economic impact

The meeting reviewed substantial evidence regarding cost-effectiveness and economic impact of new testing methods for *T. cruzi* infection and for other diseases, with key studies and data presented in Appendix 6 SI.

Group 3 discussed the different models, assumptions, and recommendations presented during the meeting. After the meeting, the group finalized the cost-effectiveness substudy, annexed to the harmonized research protocol for the evaluation of RDTs in a specific country (Appendix 7 SI). Briefly, the four components on this substudy are: (A) estimating the potential impact (effectiveness) of the current diagnostic-care-cascade and intervention scenario; (B) estimating the costs associated with the different testing algorithms from both the patient and provider-perspectives, using the Excel-based tool developed for this purpose; (C) evaluating the cost-effectiveness of the different testing algorithms (including sensitivity analyses on key parameters). The effectiveness of each algorithm and its incremental costs depend on the performance of the tests used, which is directly related to the decision to treat after diagnostic confirmation; and (D) performing a budget impact analysis (BIA) to assess the financial impact of adopting a new algorithm.

### Future perspectives and conclusions

This consensus meeting sought to merge diverse and often contradictory evidence from multiple disciplines bringing together scientists and technical authorities, and diagnostic experts on *T.cruzi infection* from around the globe.

As evidence with varying quality and performance for new assays becomes available across the region, it is important to demonstrate their suitability for global public health needs. Variability may arise not only from the pathogen’s genetic diversity and disease complexity but also from differences in testing standards.

We achieved consensus on harmonized evaluation strategies for RDTs, key product characteristics for molecular tests, and standards for evaluation, as well as contributing to PAHO guidelines in RDT uses in order to advance in a more harmonized way to diagnosis decentralization.

### Facilitating robust scientific outputs for new diagnostics

We recognize that the harmonized protocol to evaluating RDTs is generic and may not capture local scenarios or cross-country variations. However, experts focused on reaching consensus to produce globally applicable estimates valid for all settings and use cases.

Our discussion shed light on multifaceted aspects of RDTs for screening or diagnosing chronic *T.cruzi* infection. Drawing from HIV diagnosis, where the community have adapted to using multiple RDTs for diagnosis, experts emphasized the need for evidence on RDTs-based diagnostic algorithms. for diagnosis for hard-to-reach populations, especially in regions where evidence on individual RDT performance with their autochthonous populations is available (Bolivia and Paraguay).

Given the concerns raised by the experts about the limited evidence generated in some regions, and the presence of low-reactivity samples in Guatemala and Mexico, it is important to support the implementation of new studies in these regions.

Although national-level RDT implementation studies have limitations, emerging data suggest that laboratory validation studies yield results comparable to field validations. Thus, lab-based studies offer a faster, lower-cost strategy for test validation in resource-limited settings.

### Paving the way for access to quality-assured, affordable and equitable diagnostics

Experts underlined the accelerated development of innovative diagnostics from near POC (basic laboratories) to true POC (portable, battery-operated or instrument-free) during the COVID-19 pandemic. We initiated consensus discussions on key product characteristics for “priority” and “transformative” diagnostics, to guide future approaches for diagnosing acute and vertically transmitted *T. cruzi* infection.

It was also specified that test parameters were key for other use cases, such as oral infections or CD reactivation (coexistence with HIV and AIDS). Because their detection with high-performing molecular tests is now prioritized in some countries (like Brazil), given that they present a greater risk and are a more recurring phenomenon due to climate change and urbanization.

Despite progress, major gaps in *T.cruzi* diagnostics remain. While some incremental advances may address needs in specific epidemiological or geographic settings, others risk redundancy. Our conclusions provide a robust rationale for funding proposals aimed at developing transformative diagnostic tools or supporting country-specific validation studies for priority diagnostic methods.

### Priority setting to advance in *T. cruzi* POC diagnostics

#### Health policy and collaboration

- Global collaboration is crucial to addressing this NTD. However, challenges and hazards change rapidly: pathogens evolve, outbreaks escalate into epidemics/pandemics, and novel technologies become more popular. Methodologies must keep pace with the state of the scientific progress.
- Diagnostics development often requires large investments, with scarce resources available. It is critical that WHO, buyers/procurers of tests (e.g. national authorities and PAHO) and end-users (providers and patients), must clearly communicate their needs for fit-for-purpose products and actively guide their development and evaluation. We recommend that these key stakeholders are convened and collaborate with industry (public and private) partners to update a Target Product Profile (TPP) for *T. cruzi* infection POC diagnostics. Particularly, reaching consensus on test prices (for reagents only and end-users), instrument prices, and scale of manufacture needed, to inform public, private, and philanthropic investors— and attract new investors—to fund those candidate products likely to have the largest public health benefits.
- Discussions highlighted the significance of health diplomacy and strategic positioning of PAHO and WHO to propel the production of accessible materials for researchers (reference laboratory standards, quality control panels, circulating isolates) and developing protocols for their regional manufacturing.

#### Incentives and financing for R&D and for improving access

- The pricing of new diagnostics exhibits significant variability between countries, with low demand for certain tests posing challenges to supply continuity, particularly when dominated by single manufacturers in high-income countries. Addressing this issue requires solutions that extend beyond financial considerations. Key strategies should include defining and promoting regional manufacturing capabilities while incentivizing pooled procurement of diagnostics through mechanisms like the PAHO Strategic Fund. Such procurement could be implemented alongside mainstream diagnostics for conditions such as perinatal HIV, syphilis, and hepatitis B, or integrated with diagnostic tools for other neglected tropical diseases.
- For low-volume products, where pooled procurement proves unfeasible, alternative R&D and access models must be explored to ensure affordability while maintaining incentives for innovation. These could include technology transfer agreements and negotiated access terms with developers and manufacturers. While convincing traditional manufacturers to produce small batches for affected countries presents challenges, engaging small and medium-sized enterprise manufacturers capable of small-batch production offers a viable solution. Systematic collaboration with these manufacturers and test developers should be pursued, leveraging their interest in establishing credibility with health authorities.
- Closing the large financing gap will require a major effort to mobilize new resources. Particularly, governments in endemic countries must engage further, as they are under-performing in their overall contribution to R&D *T. cruzi* diagnostics, given their economic capacity (many of them upper-middle-income) and their burden of disease [17]. Product Development Partnerships and their funders should continue investing in R&D, while innovative access models are explored. Additionally, as CD remains a global public health concern, high-income governments can play a crucial role in financing diagnostic development.

#### Democratization of information

- Exposing new projects and products to early scrutiny can help prevent wasting valuable resources and enhance the signal-to-noise ratio in the overall scientific literature. Therefore, there is a clear and pressing need to create more robust platforms for facilitating the distribution and accessibility of knowledge among all key stakeholders.
- Accordingly, we propose the creation of an international task force, endorsed by PAHO and WHO, dedicated to collecting, elaborating and centralizing information, evaluation frameworks, and tools applicable for development, standardization, validation and implementation of new testing methods for CD. This task force would convene periodically to actively *(i)* identify critical remaining diagnostic gaps, sharing regional challenges or global priorities; *(ii)* disseminate efforts and recommendations to promote integration, and funding optimization; and *(iii)* raise awareness and engagement among at-risk communities, contributing to prompt detection and response strategies.

## Electronic supplementary material

Below is the link to the electronic supplementary material.

Supplementary Material 1

## Abbreviations

CD: Chagas disease
POC: Point-of-care
RDT: Rapid diagnostic test
PAHO: Pan American Health Organization
WHO: World Health Organization
NTD: Neglected Tropical Disease
LAMP: Loop-mediated isothermal amplification
qPCR: Real-time quantitative PCR
PCR: Polymerase chain reaction
DTU: Discrete Typing Unit
Se: Sensitivity
Sp: Specificity
CI: Confidence interval
BIA: Budget impact analysis
QA: Quality assurance
TPP: Target Product Profile
R&D: Research and development
HIV: Human immunodeficiency virus
AIDS: Acquired immunodeficiency syndrome
DNA: Deoxyribonucleic acid
WB: Whole blood
DBS: Dried blood spots
GE: Guanidine Hydrochloride + EDTA (buffer solution)
EDTA: Ethylenediaminetetraacetic acid
USD: United States Dollar
INS: Instituto Nacional de Salud (Chile)
FIND: Foundation for Innovative New Diagnostics
DNDi: Drugs for Neglected Diseases initiative
INGEBI-CONICET: Instituto de Investigaciones en Ingeniería Genética y Biología Molecular – Consejo Nacional de Investigaciones Científicas y Técnicas
UFCSPA: Federal University of Health Sciences of Porto Alegre
CIBERINFEC: CIBER de Enfermedades Infecciosas
ISCIII: Instituto de Salud Carlos III
SI: Supplementary Information
