# Supplementary material for: Advancing diagnostics for Chagas disease: key product characteristics and harmonized evaluation strategies - an expert meeting report
Source: BMC Infect Dis. 2026 Jun 17;26(Suppl 1):1160. doi: 10.1186/s12879-026-13565-3 (PMC13276932; doi:10.1186/s12879-026-13565-3)
Supplement: Supplementary file 1 — Supplementary Material 1 [file 12879_2026_13565_MOESM1_ESM.docx]

**Supplementary Material**

**Title:** Advancing diagnostics for Chagas disease: key product characteristics and harmonized evaluation strategies - An expert meeting report

**Authors**: Laura C. Bohorquez, Alejandro G. Schijman, Freddy Perez, Hector Coto, Andrea Marchiol, Maria-Jesus Pinazo

**Supplementary appendix**

**Table of Contents**

Appendix 1. List of attendees. Page 2

Appendix 2. Additional results related to the harmonized protocol to evaluate RDTs. Page 6

Appendix 3. Guidance on sample size estimations in the harmonized protocol to evaluate RDTs. Page 7

Appendix 4. Molecular testing of *T. cruzi* and challenges in development and evaluation. Page 8

Appendix 5. Summary of pre-meeting survey results about the key product characteristics of Real Time PCR tests. Page 9

Appendix 6. Evidence on cost-effectiveness and economic impact of new testing methods. Page 10

Appendix 7. Annex to Generic Protocol: Cost-effectiveness sub-study (version 06 June 2024). Page 11

References. Page 17

Chagas cost data collection workbook (excel). Annex to Generic Protocol: Cost-effectiveness sub-study (version 06 June 2024)

**Appendix 1. List of attendees**

**Day 1**

| Name | Affiliation (country) |
| --- | --- |
| Laura Bohorquez # * | FIND (Colombia) |
| Shaukat Khan # | FIND (Switzerland) |
| Kyra Grantz # | FIND (Switzerland) |
| Sarah Girdwood # | FIND (Switzerland) |
| Marcelo Rodriguez | FIND (Argentina) |
| Alejandro Schijman # * | INGEBI-CONICET (Argentina) |
| Arturo Muñoz | INGEBI-CONICET (Argentina) |
| Maria Jesus Pinazo * | DNDi (Brazil) |
| Natalie El Kheir | DNDi (Brazil) |
| Andres Caicedo # | DNDi (Brazil) |
| Rafael Herazo # | DNDi (Brazil) |
| Colin Forsyth * | DNDi (USA) |
| Freddy Pérez * | PAHO (USA) |
| Hector Coto | PAHO (USA) |
| María Isabel Jercic # | INS (Chile) |
| Belkisyolé Alarcón de Noya | Intituto de Medicina Tropical-UCV (Venezuela) |
| Alejandro Hasslocher | Fiocruz (Brazil) |
| Andrea García Balderrama | INLASA (Bolivia) |
| Antonieta Rojas de Arias | CEDIC (Paraguay) |
| Constança Britto * | Fiocruz (Brazil) |
| Franciana Rosa Da Silva | CUIDA Chagas (Brazil) |
| Fred Luciano Neves Santos | Fiocruz (Brazil) |
| Igor Almeida | UTEP (USA) |
| Julio Alonso Padilla * | ISGlobal (Spain) |
| Karina Egüez Soliz | Servicio Departamental de Salud - Ministry of Health (Bolivia) |
| Karla Lange | Universidad de San Carlos de Guatemala (Guatemala) |
| Laura Lamfre | redArets (Argentina) |
| Lizeth Rojas Panozo | CEADES (Bolivia) |
| Oscar Noya | Intituto de Medicina Tropical-UCV (Venezuela) |
| Otacilio Moreira # | Fiocruz (Brazil) |
| Santiago Hasdeu # | redArets (Argentina) |
| Vidalia Lesmo | SENEPA (Paraguay) |
| Yerly Magnolia Useche # | CUIDA Chagas (Brazil) |
| Zulma Cucunuba | Universidad Javeriana (Colombia) |
| Bertha Espinoza | IIBO-UNAM (Mexico) |
| Montserrat López Serafín | INDRE (Mexico) |
| Fernan Aguero | IIBio-UNSAM-CONICET (Argentina) |
| Jaime Altcheh | Hospital De Niños Ricardo Gutierrez (Argentina) |
| Marcelo Abril | Fundación Mundo Sano (Argentina) |
| Margarita Bisio * | INP Fatala Chaben (Argentina) |
| Nasim Iusef | Ministerio De Salud Provincia De Buenos Aires (Argentina) |
| Silvia Longhi | INGEBI-CONICET (Argentina) |
| Juan Carlos Ramirez | Hospital De Niños Ricardo Gutierrez (Argentina) |
| Rocío Rivero | INP Fatala Chaben (Argentina) |
| *Presentations (virtual)* |  |
| *Elena Ivanova #* | *FIND (Switzerland - virtual)* |
| *Elisa Sicuri #* | *ISGLOBAL (Spain - virtual)* |
| *Andrea Marchiol #* | DNDi (Brazil) |

# Speakers, * Moderators

**Day 2**

GROUP 1. Harmonized protocol for the evaluation of RDTs

| Name | Affiliation (country) |
| --- | --- |
| Laura Bohorquez * | FIND (Colombia) |
| Andres Caicedo | DNDi (Brazil) |
| Rafael Herazo | DNDi (Brazil) |
| Freddy Pérez | PAHO (USA) |
| Hector Coto | PAHO (USA) |
| María Isabel Jercic * | INS (Chile) |
| Alejandro Hasslocher | Fiocruz (Brazil) |
| Andrea García Balderrama | INLASA (Bolivia) |
| Franciana Rosa Da Silva | CUIDA Chagas (Brazil) |
| Fred Luciano Neves Santos | Fiocruz (Brazil) |
| Karina Egüez Soliz | Servicio Departamental de Salud - Ministry of Health (Bolivia) |
| Karla Lange | Universidad de San Carlos de Guatemala (Guatemala) |
| Oscar Noya | Intituto de Medicina Tropical-UCV (Venezuela) |
| Vidalia Lesmo | SENEPA (Paraguay) |
| Bertha Espinoza | IIBO-UNAM (Mexico) |
| Fernan Aguero | IIBio-UNSAM-CONICET (Argentina) |
| Jaime Altcheh | Hospital De Niños Ricardo Gutierrez (Argentina) |
| Silvia Longhi # | INGEBI-CONICET (Argentina) |
| Rocío Rivero | INP Fatala Chaben (Argentina) |

* Moderators # Relator

GROUP 2. Key product characteristics and quality standards of molecular tests

| Name | Affiliation (country) |
| --- | --- |
| Marcelo Rodriguez | FIND (Argentina) |
| Alejandro G. Schijman * | INGEBI-CONICET (Argentina) |
| Arturo Muñoz # | INGEBI-CONICET (Argentina) |
| Maria Jesus Pinazo | DNDi (Brazil) |
| Colin Forsyth | DNDi (USA) |
| Belkisyolé Alarcón de Noya | Intituto de Medicina Tropical-UCV (Venezuela) |
| Antonieta Rojas de Arias | CEDIC (Paraguay) |
| Constança Britto | Fiocruz (Brazil) |
| Igor Almeida | UTEP (USA) |
| Julio Alonso Padilla | ISGlobal (Spain) |
| Lizeth Rojas Panozo | CEADES (Bolivia) |
| Otacilio Moreira * | Fiocruz (Brazil) |
| Montserrat López Serafín | INDRE (Mexico) |
| Margarita Bisio | INP Fatala Chaben (Argentina) |
| Juan Carlos Ramirez | Hospital De Niños Ricardo Gutierrez (Argentina) |

* Moderators # Relator

GROUP 3. Cost-effectiveness and economic impact of new testing methods

| Name | Affiliation (country) |
| --- | --- |
| Shaukat Khan * | FIND (Switzerland) |
| Kyra Grantz # | FIND (Switzerland) |
| Sarah Girdwood * | FIND (Switzerland) |
| Natalie El Kheir # | DNDi (Brazil) |
| Freddy Pérez | PAHO (USA) |
| Laura Lamfre | redArets (Argentina) |
| Santiago Hasdeu | redArets (Argentina) |
| Yerly Magnolia Useche | CUIDA Chagas (Brazil) |
| Zulma Cucunuba | Universidad Javeriana (Colombia) |
| Nasim Iusef | Ministerio De Salud Provincia De Buenos Aires (Argentina) |

* Moderators # Relator

**Appendix 2.** Additional results related to the harmonized protocol to evaluate RDTs (refer to **Table 1** in the main text)

| Pre-meeting Survey | | During the meeting |
| --- | --- | --- |
| Consensus  (>50% agree) | **Statement *(Do you agree on including / changing in the protocol the following?*)** | **Conclusions of Plenary Discussions and Consensus Reached by Group 1** |
| And <15% neutral or disagree | **Structure** | **Purpose.** Distinguish the evaluation of RDTs intended either for screening or for diagnosis confirmation.  **Outline** **inclusion/exclusion criteria for samples/patients**. Exclude patients who had been treated with antiparasitic drugs.  **Informed Consent**. Translated to local languages (indigenous populations), adapted to illiterate people, and assent released for minors and children. |
|  | **Expected predictive values.** At different prevalence (<5%; 5-10%; >10%) as test acceptance criteria | Se/Sp are prevalence-independent characteristics, but decisions on public health depend on prevalence. It is necessary to analyze the predictive values to understand the coverage margin of the policies to be implemented. Experts did not reach consensus, as there is not yet sufficient data in the region. |
|  | **Guidance on proper results recording** | RDT results are classified as positive/reactive, negative/non-reactive, or invalid as per manufacturer’s instructions (remove the "indeterminate" classification for RDT results).  Conduct standardized photo results recording. Include more than one RDT reader for interpretation. |
|  | **Secondary objectives** | May provide assessing as secondary objective the cost-effectiveness to accompany the conclusions (task assigned to Group 3), and ease-of-use of the investigational products. |
| And 15-32% neutral or disagree | **Investigational Products (RDTs)** | Do not recommend selecting RDTs with different antigens for the evaluation. This information is not always available, and local validation studies set the actual performance in the country of interest. |
|  | **Quality Assurance** | Offer accessing certified external panels, e.g. WHO International Standard anti-*T.cruzi* I and II Antibody Reference Panel. |

**Appendix 3. Guidance on sample size estimations in the harmonized protocol to evaluate RDTs**

Consensus was reached on the guidance on sample size estimations, to be included the harmonized protocol to evaluate RDTs. Recommend adding the reference to the book that has been widely cited on diagnostic evaluations (1), to calculate the number of confirmed positive/negatives by the reference test method, needed to estimate expected sensitivity/specificity with the given CI **(Table 1).** If researchers would like to adjust the parameters, they can use the shinyapp developed by FIND, publicly available <https://finddx.shinyapps.io/SampleSize/>. In brief, the formula is as follows:

$$n= \frac{[(Z_{\frac{\alpha}{2}}+ Z_{\beta})\sqrt{V{(\theta)}^{2}}}{{(L)}^{2}}$$

Where Za/2 is the upper a/2 percentile of a standard normal distribution, Ζβ is the upper β percentile of a standard normal distribution where 1- β is the desired power, and L is the desired width of one-half of the CI. And the formula which links the n to be screened to statistical power, based on the prevalence:

$$\frac{\left( N_{total} \times{Prev}_{p} \right)-n}{\sqrt{{N_{total} \times Prev}_{p} \times(1-{Prev}_{p})}} = Z_{\beta}$$

Where Prevp is the prevalence, and n is the n from the calculation above.

**Table 1.** Sample size needed to estimate sensitivity/specificity with the given CI, with 80% power and 5% significance level

| Sensitivity / Specificity (expected for index test) | Error Margin (half width CI) | Disease Prevalence | n Positives /  n Negatives | N Total to Screen |
| --- | --- | --- | --- | --- |
| 95% | 2.50% | 5% | 597 | 12338 |
| 98% | 2.50% | 5% | 247 | 5188 |
| 95% | 2.50% | 10% | 597 | 6164 |
| 98% | 2.50% | 10% | 247 | 2590 |
| 95% | 2.50% | 15% | 597 | 4106 |
| 98% | 2.50% | 15% | 247 | 1725 |
| 95% | 2.50% | 20% | 597 | 3076 |
| 98% | 2.50% | 20% | 247 | 1292 |

For precision, recommended to work with a maximum width of the 95% CI of 5% (+/- 2.5%). If researchers prefer to reduce the sample size, or if it is not feasible to perform the study with such a sample size, first consider decreasing the statistical power (from 90 to 80%). Secondarily, widening the CI. Estimations should be reviewed by a statistician who tailors to the specific study objectives.

**Appendix 4. Molecular testing of *T. cruzi* and challenges in development and evaluation**

| Category | Key Characteristics / Trade-offs |
| --- | --- |
| *Available Technologies:* | |
| LAMP (late development - validation) | Need for standardizing POC DNA extraction methods at or near the site of patient care, ideally compatible with qPCR, including (1) a repositioned 3D printer to rapid DNA extractor (AI Biosciences, USA), and (2) an ultrarapid DNA extraction method (PURE-Eiken Chemical Co, Japan).  Need for portable equipment remains a significant limitation for field use.  Clinical Sp reported compared to RT-PCR for detection of vertical infection was 98.6% for neonates at birth, and 98.2% at 2 months of age (2). |
| Real Time PCR (commercial kits) | Consensus agreement to evaluate qPCR methods for detection of *T. cruzi* DNA available in (3,4), and Target Product Profiles (TPPs), in 2015, ﻿for POC diagnosis based on evidence available up to 2011, and in 2020 ﻿for assessment of treatment efficacy in CD patients (5,6).  Some kits allow collecting the blood of patients in hard-to-reach areas, mixing it with guanidine hidrochoride 6M, EDTA 0.2 M, pH 8.00 buffer, and transporting it, without a cold-chain, to central laboratories.  Performance exhibited in a head-to-head comparison of four qPCR kits, showed that two had all lower bounds (95% CI) of Se, Sp, and predictive values ≥96.4%. |
| *New molecular POC diagnostics that could be adapted to detect T. cruzi and have a transformational impact in primary healthcare facilities:* | |
| Development Pipeline | 161 total molecular diagnostic tools, 10 supported by FIND  10 true POC tests  3 instrument-free options |
| PCR assays with reader | High power requirements, higher costs |
| Isothermal assays with reader | Lower sensitivity, limited multiplexing capacity |
| Isothermal single-use platforms | Higher cost per test, environmental impact concerns |
| Novel methodologies | Early-stage technology, limited clinical data (other diseases) |
| Key Technologies Summarized | Isothermal amplification techniques, amplification-free methodologies |
| Challenges in development for *T. cruzi* detection | **1. Limited diagnostics menu.** Requiring additional financing and incentives  **2. Lack of updated TPP.** Tailored to the current needs and technology, developed by the health authorities, end users and industry  **3. Potential sample incompatibility** (whole blood/urine) |
| Issues for the evaluation | **1. Access biospecimens.** Requiring collaborations and setting the requirements (calibration curves, interlaboratory studies to reduce bias, and amplification curves to increase precision)  **2. Lack of international reference standards.** Hindering comparison of parasitic loads between laboratories  **3. Lack of (quantitative) positive controls and** **panels** for verification, validation, and performance evaluation |

**Appendix 5. Summary of pre-meeting survey results about the key product characteristics of Real Time-PCR tests**

| Consensus  (>50% agree) | Product Characteristic | Minimum Requirement | Ideal Requirement |
| --- | --- | --- | --- |
| <15% neutral or disagree | Target Analyte | *T. cruzi* DNA and internal amplification control | Multiplex |
|  | Reference Method | Microscopy / Standard algorithm for vertical infection | |
|  | Analytical Specificity | No cross-reactivity with other *Trypanosoma spp., Leishmania spp*., or other pathogens | |
|  | Strain Specificity | Detects all DTUs | |
|  | Quantitation | No | Yes |
|  | Training Needs | Compartmentalized rooms for PCR laboratory (PHS, 2017 Q4E guide). One-week training for a technician / biochemist with advanced skills in molecular techniques | |
|  | Specimen Type | Anticoagulated blood with stabilizing agent (e.g. Guanidine Hydrochloride EDTA) | Anticoagulated blood without stabilizing agent (GE) DBS |
|  | Time sample collected to result | Two days | One day |
|  | Internal Quality Control | Positive control, non-template control, and negative DNA extraction control included in kit | Quantitative positive control included in kit |
|  | External Quality Control | Standard (third-party) reference panels | International certified harmonized (third-party) panels. And prospective field studies with blind samples |
| 16-28% neutral or disagree | Intended use | Diagnosis for patients suspected of acute infection (all transmission modes or infection reactivation) | Diagnosis for patients suspected of acute infection (all transmission modes or infection reactivation); Asymptomatic / symptomatic patients suspected of chronic infection; Assessment of response to antiparasitic treatment in chronic phase |
|  | Diagnostic Sensitivity | ≥92% (point estimate with maximum +/- 5% -95%CI) | ≥95% (point estimate with maximum +/- 5% -95%CI) |
|  | Diagnostic Specificity | Comparable to microscopy | |
|  | Analytical Sensitivity | One eq. par./ml in blood | 0.1 - 0.5 eq. par./ml |
|  | Specimen prep | Column based DNA extraction commercial kit | |
|  | Reagent kit stability | 6 months | 18 months |
|  | Quality Assurance | Proficiency testing panels evaluated before starting implementation of a new assay, and every two years thereafter. | Proficiency testing panels evaluated every year. |
|  | Test Price (reagents costs, exworks) | Less than 20 USD | Less than 15 USD |
|  | Instrument Price (exworks) | Less than 40,000 USD two-channels thermocycler | Less than 25,000 USD two-channels thermocycler |

**Appendix 6. Evidence on cost-effectiveness and economic impact of new testing methods**

| Category | How and what is measured | Conclusions |
| --- | --- | --- |
| Assessing cost-effectiveness (CE) of *T. cruzi* diagnostic algorithms | Comparing CE of current *T. cruzi* diagnostic algorithm against new RDT testing algorithms in Argentina (ongoing, FIND). | - RDT diagnostic algorithm is as efficient in identifying positive cases as the current testing algorithm, but more efficient when patients visit costs were included.  - Serial algorithm versus parallel algorithms (combining 2-3 RDTs) identified slightly fewer positive cases but was cheaper per patient identified due to fewer tests being performed. |
|  | Presented an interactive online tool by FIND:  <https://finddx.shinyapps.io/chagaspathway/> | It allows non-expert users to model impact outcomes (total costs, cases linked to treatment, DALYs, predictive values, cost per disease), by choosing different diagnostic algorithm structures, and using as input different tests parameters (type, performance, and settings), costs to health system and patient, loss to follow up, prevalence, linkage to treatment and treatment effect. |
|  | Analysis using a decision tree and a Markov model, to compare CE of different testing algorithms based on RDTs and/or laboratory serology in Bolivia (under preparation for publication, *Sicuri et.al*). | Most cost-effective testing strategy was RDT + laboratory serology, for RDT sensitivity <90%.  RDT-only strategy is increasingly more cost-effective for RDT sensitivity >90%, and for lower prevalence (e.g. 1%). |
|  | Comparing CE of RDT algorithms versus current testing algorithms, in Brazil, Bolivia and Colombia. QALYs, cost-utility, discounting for costs and benefits at a rate of 3-5%, diagnosis opportunity and treatment coverage will be measured in this ongoing multi-country study (7). | A model simulation incorporates patient and societal costs perspectives, as well as CE thresholds to assess whether an intervention is worthwhile and should reflect health opportunity cost of other interventions. Cost and health consequences of interventions are discounted at a rate following guidelines for technology assessment (Brazil and WHO-CHOICE). |
| Multi-dimensional approaches | Analyses of patients-incurred costs in Colombia, as the impact of receiving care for *T.cruzi* infection in primary healthcare facilities (8). | Patient perspective showed a 4-fold reduction in travel time, a 5-fold reduction in transport expenses, 5.5-fold reduction in food and housing expenses, and a 2-fold reduction in income losses. |
|  | Budget impact analysis and CE analyses of POC molecular diagnosis of human papillomavirus and tuberculosis, in Argentina (RedARETS). | Examples not related to CD diagnostics, conducted by public health technology agencies that have influenced policy changes, emphasized the importance of including “hidden” costs (social indirect costs, instruments maintenance, lifetime cycle, spare parts, software updates, human resources needed, and their regional differences across countries). |

**Appendix 7. Annex to Generic Protocol: Cost-effectiveness sub-study (version 06 June 2024)**

**Annex to Generic Protocol: Cost-effectiveness sub-study**

**Overview**

A cost-effectiveness analysis can be conducted to compare (1) the current standard of care for diagnosing chronic *T.cruzi* infection to (2) new algorithms that incorporate new testing technologies such as rapid diagnostic tests (RDTs) adopted at lower levels of the healthcare system (the *intervention*). This is an annex to the main study, which will evaluate the performance of the new algorithms incorporating these new test technologies in Setting X.

Two main scenarios will be modelled:

***1) Standard of care (the comparator)*:** This represents the current standard of care, or status quo, for diagnosing chronic *T.cruzi* infection in Setting X. The standard of care can be described, detailing the sample type, test technology, algorithm structure and the place of testing. For example, venous blood is collected from individuals accessing high complexity centres (secondary/tertiary centres), or samples are transported to high- complexity centres from low complexity centres (primary healthcare centres) for testing. Two laboratory-based serology IgG tests for *T. cruzi* are performed in parallel; a third test is performed in case of discordance.

***2) Intervention*** (***the new algorithms incorporating new testing technologies*):** This is the new proposed algorithms that include RDTs which can be used to decentralize the diagnosis of *T. cruzi* to lower levels of the healthcare system.

There are four components to this study: (A) Estimating the potential impact in terms of effectiveness of the Chagas disease (CD) diagnostic care cascade in relation to the standard of care and intervention scenario, (B) estimating the costs associated with the different testing algorithms, (C) evaluating the cost-effectiveness of the different testing algorithms, and (D) performing a Budget Impact Analysis (BIA) to assess the financial impact of adopting a new algorithm.

The approach described in this Annex is conservative in terms of determining the full benefit of the introduction of a new technology to diagnose chronic *T.cruzi* infection. It only incorporates the direct benefits in the diagnostic pathway for CD, however, there are potentially many additional benefits that are likely to arise that strengthen the primary healthcare system more broadly. These however are not quantified here. More complex analyses may be conducted that measure improvements in access for those seeking care or conduct a distributional cost-effectiveness analysis that evaluates how the intervention improves health inequalities for different population sub-groups.

**Chagas Diagnostic Cascade Model Framework**

The performance and impact of the different diagnostic cascades may be directly estimated from primary data from evaluation or pilot studies that assess outcomes among patient groups tested under different algorithms. In these situations, studies should be designed to collect appropriate data to estimate the **key outcomes** listed below, ensuring the representativeness of the study population for the target population of interest.

Where it is not possible to directly measure the **key outcomes** of each diagnostic algorithm of interest, modelling, using secondary data sources, can be a useful tool to estimate the overall impact of diagnostic algorithms based on data from individual test performance. A model of the CD diagnostic care cascade in Setting X can be created using the FIND Chagas Diagnostic Algorithm application (https://finddx.shinyapps.io/chagaspathway/) developed with the support of DNDi, or alternative decision-tree models. Models representing the current standard of care within Setting X should be compared to models representing the diagnostic algorithms incorporating new testing technologies to estimate changes in overall diagnostic accuracy and costs.

An example of a generic diagnostic care cascade for standard of care and a new algorithm that incorporates RDTs at a lower level of care, is shown below.

***Figure 1. The Chagas disease diagnostic cascade***

These decision tree models should capture the diagnostic tests used, the performance (sensitivity/specificity) of these tests and the healthcare level at which patients seek care and at which samples are collected and tested. The patient and sample flow should be considered to identify touchpoints at which individuals or samples could be lost from the cascade and estimate the number of visits required to receive a final diagnosis. For both scenarios (standard of care and the intervention), an estimated number of individuals diagnosed correctly with *T.cruzi* infection (of those who truly have *T.cruzi* infection) is determined as well as the number of individuals who receive the positive test(s) who are successfully linked to further care/treatment.

The new intervention algorithms will then be compared to the standard of care against a number of **key outcomes** which seek to capture the potential for increased yield through better test performance or increases in accessibility to testing and results, or a reduced proportion of individuals being lost during the diagnostic process with new diagnostic algorithms:

The number of individuals receiving a correct diagnosis (true positives/negatives)

The number of individuals receiving an incorrect diagnosis (false positives/negatives)

The number (and proportion) of positive individuals linked to further care/treatment

The number of each test type conducted

The number of patient visits (by healthcare level) prior to diagnosis

Number of individuals lost to follow-up prior to final diagnosis

Additional: Disability-adjusted life-years (DALYs) averted through linkage to treatment

Parameters required for the Chagas Diagnostic Algorithm application to be estimated through the main Performance Evaluation Study, as well as possible sources for parameters that are not collected through evaluation studies, are described in Table 1. Note that parameter values should be customized to represent the specific context and population of interest.

The end-point for this analysis is the confirmation of the final diagnosis and linkage to further care/treatment. The downstream impact of treatment is not included. Should the end-point include treatment of those diagnosed with *T.cruzi* infection, additional parameters/data are required and an additional model (e.g. Markov model) is necessary to evaluate the impact of treatment. For example, outputs on the number of cases linked to further care/treatment from the FIND Chagas Diagnostic Algorithm application can be used as inputs into a disease progression model or other to estimate the downstream impact.

***Table 1. Required parameters for CD diagnostic cascade model***

| **Cost category** | **Detail** |
| --- | --- |
| Population | Seroprevalence of *T.cruzi* infection in population^[[1]](#footnote-1)^ being tested as part of this study (prospective evaluation of RDTs for *Trypanosoma cruzi* infection in Setting X)  Socio-demographic characteristics of those being offered the test: age, sex, urban/rural, access to healthcare, level of education (as aligned with main protocol and the sub-analyses stipulated there).  *Source: surveillance data; recruitment participant data* |
| Test characteristics | Test assay sensitivity, specificity  *Source: literature/manufacturer specifications, performance evaluation study* |
| Linkage to further care/treatment | Probability linked to further care/treatment following a positive test result. This can differ depending on the setting where the final diagnosis is made, the target population and the availability of treatment at this healthcare level.  *Source: literature* |
| Loss to follow-up | Loss to follow-up in the diagnostic cascade occurs whenever a patient has to return for a subsequent visit prior to the final diagnosis confirmation or where a sample has to be transported to another facility for processing and testing. It is the percent of the population seeking care who are unlikely to return for an additional visit – alternatively, the probability that a sample is lost between collection and testing.  *Source: literature, National surveillance system, as per defined standard definitions : for example – no contact for more than 30 days.* |
| Access | Estimated increase in the population who will be able to access testing (for example, due to decentralized RDT testing).  *Source: literature, to be varied in a sensitivity analysis* |
| Error rates | Proportion of test results that are expected to be invalid/indeterminate (and require a second test of that test type)  *Source: performance evaluation study, manufacturer specifications* |

**Cost analysis**

The fully loaded testing cost per individual tested for *T.cruzi* infection will be estimated for the intervention (the novel testing algorithms) and the standard of care. An ingredients-based approach will be used to identify and quantify all the inputs required to perform the respective test, as well as their estimated quantities and value. Costs will reflect both the **patient- and provider-perspective**. Costs will be estimated on a per test level, and on a per healthcare visit level for both the patient and the provider.

***1) Test-level***: This includes the cost of the test kit/reagents, equipment and consumables required to conduct a given test, cost to transport a sample, as well as the staff salary cost associated with time spent on sample collection, testing, and interpreting the test result by the relevant healthcare-worker cadre at different levels of care. Additional costs relating to training, quality assurance etc, are detailed in Table 2 below and the accompanying workbook on data collection.

***2) Patient-level visit cost:*** A patient-level visit cost will be assigned to the number of visits that it takes an individual to receive a diagnosis, by level of care accessed. This is the cost to the patient of visiting a healthcare centre and may include transportation, accommodation, food, and other non-medical out-of-pocket costs, as well as productivity loss costs associated with the time spent seeking care. The cost to the patient may differ depending on the level of the healthcare system that the patient accesses – for example, higher costs may be incurred to access high-complexity centres versus low-complexity centre. These costs may be sourced from the literature or estimated as part of a separate study (see cost workbook for additional details).

***3) Provider-level outpatient visit cost***: An outpatient visit cost is the overhead cost borne by the health care system associated with a patient visit. The cost is differentiated by the different levels of the healthcare system where the individual is seeking care and receiving testing: e.g. low complexity centres versus high complexity centres. The cost includes overhead costs such as utilities, infrastructure/space costs, staff overhead costs etc. and can be collected as part of the study or estimated using the literature.

The cost collection workbook details the key input cost categories: staff, test consumables, test equipment, overhead costs, and transport, and visit costs to the patient and the provider. The per-test costs include all costs related to specimen collection, sample processing and analysis, data management and result delivery. Resource use will be determined through interviews with individuals involved in the implementation of the performance evaluation study in Setting X. All costs will be reported in YEAR USD and converted using standard market rates from the local currency in Setting X. All capital costs will be annuitized and discounted using the discount rate most appropriate for Setting X.

**Cost-effectiveness analysis**

Costs, as described above, will be assigned to resource outputs (number of tests by type and location of testing, and number of individual visits before diagnosis by location) from the key outcomes of the different diagnostic algorithms (the standard of care and the intervention). Effectiveness outcomes (as described above in section A) such as the number of true positive and true negative cases, as well as the number of positive individuals linked to further care/treatment will be used to calculate the **cost per correct diagnosis** and the **cost per positive case linked to further care and treatment** for the different algorithms. The costs and the outcomes for each algorithm can then be used to calculate the incremental cost-effectiveness ratios (ICER) for each diagnostic algorithm, which compares the additional cost of one algorithm relative to the next least costly algorithm, or the standard of care. The ICER will identify the most efficient algorithm or the one that provides the greatest value for money. This formula is depicted below for Outcome^i^ - correct diagnosis, or a positive case linked to further care and treatment.

$$ICER=\frac{{Costs}_{ITV}^{i}-{Costs}_{SOC}^{i}}{{Outcome}_{ITV}^{i}-{Outcome}_{SOC}^{i}}$$

The **time horizon** is from the point where a person first seeks care to linkage to further care and treatment. No downstream costs and outcomes of treatment are included since the primary objective of this analysis is to assess the performance of alternative diagnostic algorithms and the efficacy of treatment is uncertain and the probability of a false-positive result commencing treatment is low/uncertain. However, the time horizon can be extended to include treatment outcomes and costs in an extended analysis as described in Box 1.

**Sensitivity analyses**

A one-way sensitivity analysis can be conducted on key parameters that significantly influence which algorithm is considered more cost-effective. If appropriate, a probabilistic sensitivity analysis can be conducted to demonstrate the uncertainty surrounding the cost-effectiveness results by incorporating probability distributions for the key parameters mentioned above and running multiple simulations to generate a distribution of ICERs. This can be used to construct a Cost-Effectiveness-Acceptability-Curve for different values of the cost-effectiveness threshold. If outcomes are expressed as utilities (e.g. DALYs), cost effectiveness thresholds from the literature on the opportunity-cost based thresholds can be used.

**Budget impact analysis**

A BIA aims to assess the financial implications of implementing the new diagnostic approach (the intervention) compared to the standard of care. This analysis entails determining the total cost of testing the eligible care-seeking population under both scenarios: using the standard diagnostic procedure and employing the intervention algorithm. The BIA is calculated from the payer’s perspective (depending on the setting, this is likely to be the government body who is responsible for public sector healthcare budget). The costs involved include those associated with determining the test and provider visit costs, as described above. It excludes the patient visit costs as the perspective is the healthcare funder. Care must be taken to only include *un*discounted costs. By comparing the total costs between the standard of care and the intervention algorithm, the BIA will ascertain the change in budget required for adopting the new diagnostic approach.

To conduct this analysis, the following information is required:

**Time horizon**: A time horizon of 1- 3 years is recommended.

**Eligible population**: It is necessary to estimate the *annual* care-seeking population in Setting X for which the payer is responsible (the population that is covered by the payer). For example, only those accessing care at public health facilities if the payer is a public health entity. Next, of those seeking care, estimate the eligible population for testing (those who meet the clinical criteria to be tested for CD; note this will differ by specific sub-populations, e.g. mandatory testing for pregnant women). If relevant, estimate the location (level of the health system) at which the eligible population first seeks care.

**Number of tests required per eligible individual**. Using output from the cost-effectiveness model, determine the average number of tests required per person tested taking into account loss to follow-up, level of care at which testing occurs and the algorithm structure (for example, additional tests required if there is discordance).

**Uptake of the new intervention**: To determine what proportion will receive the new intervention, it is important to estimate the uptake of the new intervention, whether this is likely to (1) completely replace the current standard of care (substitution), or, (2) there will be a combination of both the standard of care and the new intervention (for example, standard of care remains for those first seeking care at high complexity centres, and the intervention for those seeking care at low complexity centres); or, 3) the new intervention would be used at all sites where there is currently no standard of care testing or increase access to testing to the eligible population (expansion).

There are many dependencies associated with the BIA that rely on uncertain assumptions. A sensitivity analysis should explore these: for example, changes in exchange rates, inflation rates and the expected prices of the new testing technology over time, as well as the expected prices of the new testing technology may change depending on total volumes required for the different uptake scenarios. In addition, the sensitivity analysis should explore the impact of different assumptions regarding uptake of the new intervention.

This assessment will provide stakeholders, such as healthcare providers, policymakers, and payers, with valuable insights into the financial impact of implementing the new algorithm, aiding in decision-making regarding resource allocation and healthcare budget planning.

**Acknowledgments**

FIND: Sarah Girdwood, Laura Bohorquez, Kyra Grantz, Shaukat Khan

PAHO: Freddy Perez

RedARETS: Laura Lamfre, Santiago Hasdeu

LSHTM: Natalie el Kheir

**References**

1. Zhou XH, McClish DK, Obuchowski NA. Statistical Methods in Diagnostic Medicine. John Wiley & Sons; 2009.

2. Rojas Panozo L, Rivera Nina S, Wehrendt DP, Casellas A, Pinto L, Mendez S, et al. Evaluation and validation of a PrintrLab-based LAMP assay to identify Trypanosoma cruzi in newborns in Bolivia: a proof-of-concept study. The Lancet Microbe. 2024 Sep;5(9):100887.

3. Schijman AG, Bisio M, Orellana L, Sued M, Duffy T, Mejia Jaramillo AM, et al. International study to evaluate PCR methods for detection of Trypanosoma cruzi DNA in blood samples from Chagas disease patients. PLoS Negl Trop Dis. 2011 Jan;5(1):e931.

4. Ramírez JC, Cura CI, da Cruz Moreira O, Lages-Silva E, Juiz N, Velázquez E, et al. Analytical Validation of Quantitative Real-Time PCR Methods for Quantification of Trypanosoma cruzi DNA in Blood Samples from Chagas Disease Patients. J Mol Diagn. 2015 Sep;17(5):605–15.

5. Porrás AI, Yadon ZE, Altcheh J, Britto C, Chaves GC, Flevaud L, et al. Target Product Profile (TPP) for Chagas Disease Point-of-Care Diagnosis and Assessment of Response to Treatment. PLoS Negl Trop Dis. 2015 Jun;9(6):e0003697.

6. Alonso-Padilla J, Abril M, Alarcón de Noya B, Almeida IC, Angheben A, Araujo Jorge T, et al. Target product profile for a test for the early assessment of treatment efficacy in Chagas disease patients: An expert consensus. PLoS Negl Trop Dis. 2020 Apr;14(4):e0008035.

7. Sousa AS de, Vermeij D, Parra-Henao G, Lesmo V, Fernández EF, Aruni JJC, et al. The CUIDA Chagas Project: towards the elimination of congenital transmission of Chagas disease in Bolivia, Brazil, Colombia, and Paraguay. Rev Soc Bras Med Trop. 2022;55:e01712022.

8. Herazo R, Rey A, Galvão D, Camargo MM, Pinzón NJC, Sanchez ACR, et al. Analysis of the costs incurred by patients with Chagas disease: The experience in endemic municipalities in Colombia. Acta Trop. 2023 Sep;245:106963.

1. The population is as defined in the main study protocol [↑](#footnote-ref-1)
